# The selective deployment of AI in healthcare: An ethical algorithm for algorithms

**DOI:** 10.1111/bioe.13281

**Published:** 2024-03-30

**Authors:** Robert Vandersluis, Julian Savulescu

**Affiliations:** 1 Uehiro Centre for Practical Ethics, University of Oxford, Oxford, UK; 2 GSK.ai, GSK, King’s Cross, London, UK; 3 Centre for Biomedical Ethics, Yong Loo Lin School of Medicine, National University of Singapore, Singapore, Singapore

**Keywords:** algorithm, artificial intelligence, bias, exclusion, machine learning, melanoma

## Abstract

Machine-learning algorithms have the potential to revolutionise diagnostic and prognostic tasks in health care, yet algorithmic performance levels can be materially worse for subgroups that have been underrepresented in algorithmic training data. Given this epistemic deficit, the inclusion of underrepresented groups in algorithmic processes can result in harm. Yet delaying the deployment of algorithmic systems until more equitable results can be achieved would avoidably and foreseeably lead to a significant number of unnecessary deaths in well-represented populations. Faced with this dilemma between equity and utility, we draw on two case studies involving breast cancer and melanoma to argue for the selective deployment of diagnostic and prognostic tools for some well-represented groups, even if this results in the temporary exclusion of underrepresented patients from algorithmic approaches. We argue that this approach is justifiable when the inclusion of underrepresented patients would cause them to be harmed. While the context of historic injustice poses a considerable challenge for the ethical acceptability of selective algorithmic deployment strategies, we argue that, at least for the case studies addressed in this article, the issue of historic injustice is better addressed through nonalgorithmic measures, including being transparent with patients about the nature of the current epistemic deficits, providing additional services to algorithmically excluded populations, and through urgent commitments to gather additional algorithmic training data from excluded populations, paving the way for universal algorithmic deployment that is accurate for all patient groups. These commitments should be supported by regulation and, where necessary, government funding to ensure that any delays for excluded groups are kept to the minimum. We offer an ethical algorithm for algorithms—showing when to ethically delay, expedite, or selectively deploy algorithmic systems in healthcare settings.

## 1 Background

Machine-learning algorithms have the long-term potential to revolutionise diagnostic and prognostic tasks in health care. Algorithmic approaches can—at least in theory—be a more accurate, more consistent and more scalable than approaches that rely on human professionals, who are a scarce resource.^1^ Despite numerous examples of overly ambitious claims,^2^ some studies have demonstrated that algorithmic approaches have the potential to produce ‘above human’ levels of performance in diagnostic and prognostic tasks in fields such as oncology, as well as in the supporting disciplines of radiology and pathology, under a limited set of circumstances and for a narrow range of tasks.^3^ Algorithmic performance levels can, however, be materially worse for subgroups that have been underrepresented in algorithmic training data^4^—which is one of the main reasons why many impressive ‘proof of concept’ studies have failed to make it into the clinic^5^ and why many of those applications that have been deployed have come under heavy criticism.^6^

These issues have spawned a large technical literature in algorithmic bias and fairness, which has primarily focused on defining and optimising various quantitative fairness metrics.^7^ This technical literature has been complemented by a parallel philosophical literature, which has interrogated the ethical underpinnings of various fairness metrics and highlighted the inability of unidimensional fairness metrics to adequately capture complex ethical phenomena.^8^ Together, this literature helps to provide guidance as to when (and how) it can be ethically appropriate to deploy (or not to deploy) an algorithm. The key question that has been neglected is when (if ever) researchers should respond to materially worse algorithmic performance for underrepresented subgroups by immediately and selectively deploying robust algorithmic systems exclusively for better-represented subgroups. This article seeks to address this gap within the literature.

## 2 Algorithmic Deployment Options

Faced with the possibility of materially worse performance for underrepresented subgroups, system developers have three high-level options.

1. Delayed deployment: System developers can delay the release of an algorithm until it works well for all patient groups.
2. Expedited deployment: System developers can release an algorithm for all patient groups, as long as the algorithm works well for most patients.
3. Selective deployment: System developers can release an algorithm for patient subgroups where the model performs well, while withholding the algorithm from those patient subgroups for whom the model is expected to perform poorly (or unpredictably).

Delaying algorithmic deployment has the advantage of allowing more time to collect additional data from underrepresented groups, but at the cost of postponing benefits for patients who are already well represented in algorithmic training data. Alternatively, expedited deployment allows the majority of patients to enjoy the benefits of the algorithm, but at the potential cost of failing to benefit (or even inflicting harms) on underrepresented subgroups, for whom the model may not work well. Finally, selectively deploying an algorithm allows some patient groups to enjoy algorithmic benefits, while protecting excluded subpopulations from system-related harms, but at the cost of excluding these same subpopulations from the benefits of technological advancement.

Below, we will draw on two case studies to assess the ethical implications of delaying, expediting, or selectively deploying algorithmic systems. In the first case study, we will argue in favour of the selective deployment of a breast cancer prognostic model for women, even though this results in male breast cancer sufferers not receiving any immediate benefit. In a somewhat analogous case study, which involves a melanoma diagnostic model, we will explore the extent to which a selective deployment approach for light-skinned patients can be ethically acceptable, in the context of historic injustice against dark-skinned patients. From a methodological perspective, the first case study looks at the ethical obligations that are owed to underrepresented groups, while the second case study looks at how these ethical obligations are impacted when under-represented groups have also been subjected to historic injustice.

## 3 Male Breast Cancer

There are 300,000 new cases of breast cancer each year in the United States, which will result in almost 45,000 deaths.^9^ For every 100 women that suffer from breast cancer, only one man will be in the same position.^10^ The rarity of male breast cancer contributes to lower awareness levels for the condition, to later diagnoses, and to worse health outcomes for men, as compared to women.^11^ Indeed, male breast cancer sufferers are 35% more likely to die from the disease than their female counterparts.^12^ The rarity of male breast cancer also results in much more limited data, as well as complicating data collection efforts, including in clinical trials, where male patients are largely absent.^13^ A number of biological differences—including differences in hormonal profiles—make it difficult to accurately generalise from women to men in terms of treatment options and prognoses.^14^ Men and women also present breast cancer in somewhat different ways, which further complicates the diagnostic process, making it difficult to ‘bootstrap’ male insights from female data.^15^ All these factors combine to make it much less straightforward to develop diagnostic and prognostic algorithms for breast cancer in men, as compared to women.

Researchers at the University of Cambridge recently launched a prognostic algorithm for breast cancer, which leveraged health data from almost 1 million women.^16^ For all of the reasons outlined above, male patients were excluded from the modelling process, and the algorithm was selectively deployed on female patients. The Cambridge algorithm, which is extensively used by physicians and female patients alike, produces best-in-class predictions for female breast cancer sufferers with respect to various treatment options.^17^ Was the selective-deployment approach of the Cambridge researchers ethical? We argue that the answer to this question is ‘yes’, and our rationale is as follows.

First, there is a compelling ethical case for helping female breast cancer sufferers as soon as possible. Second, men are not made worse off by being excluded from model deployment. Third, men could have been made materially worse off by an expedited deployment process that sought to leverage sparse male training data, which could have also reduced the model’s performance for women. Fourth, seeking to delay the provision of urgent health benefits to large numbers of women, for the sake of ‘levelling down’ outcomes for a comparatively small number of men, would represent a disproportionate abandonment of utility for the sake of greater equity. As such, we argue that delaying the model would have treated women unethically and expediting the model for both sexes would have been harmful for men (and possibly for women) and that the selective deployment of the model for women was the most ethically appropriate option.

Due to space constraints, we will not elaborate further on our argument in favour of the selective deployment of the Cambridge prognostic model for women. Instead, we will now move to consider in greater detail the more controversial case for the selective deployment of diagnostic algorithms for light-skinned melanoma patients.

## 4 Melanoma Detection

There are several parallels that can be drawn between breast cancer in men and melanoma in patients with dark skin, as defined by the widely used Fitzpatrick scale.^18^

There are 100,000 new cases of melanoma each year in the United States, which will result in almost 8000 deaths. Melanoma is relatively rare in dark-skinned patients, when compared to light-skinned patients. For every 30 light-skinned patients that suffer from melanoma, only one dark-skinned patient will be in the same position.^19^ The rarity of dark-skinned melanoma contributes to lower awareness levels for the condition, to later diagnoses and to materially worse health outcomes for dark-skinned patients, such as many African Americans, as compared to light-skinned patients.^20^ The rarity of melanoma in dark-skinned patients also results in much more limited data sources and complicates data collection efforts, including in clinical trials, where dark-skinned melanoma patients are largely absent.^21^ Numerous biological differences—including differences in pigmentation profiles—make it difficult to accurately generalise from light- to dark-skinned patients in terms of diagnostics.^22^ Light- and dark-skinned patients also present melanoma in somewhat different ways, which further complicates the diagnostic process, making it difficult to ‘bootstrap’ dark-skinned insights from light-skinned data.^23^ All these factors combined make it much less straightforward to develop diagnostic algorithms for melanoma in dark-skinned patients, as compared to light-skinned patients.

All of the points outlined above mirror the fact pattern in the male breast cancer example, which suggests that a conclusion centring around selective algorithmic deployment would be the ethically preferable option.

Unlike male breast cancer patients, however, dark-skinned melanoma sufferers are a subgroup that has been subjected to historic injustice in terms of medical research, health outcomes, economic opportunities and life chances (among other issues). How and in what way, should a context of historic injustice influence our thinking about the ethical acceptability of selective algorithmic deployment for melanoma? We will explore this question through the DermAssist case study below.

The DermAssist case study focuses on melanoma—which is a disease that is comparatively rare for some patient subgroups, resulting in a scientific barrier to data gathering efforts. As noted in the ‘Limitations’ section below, the fact patterns for other diseases such as diabetes—where darker-skinned patients are disproportionally impacted, and there are ample opportunities to collect additional algorithmic training data—raises different ethical questions, which ought to be urgently addressed.

## 5 The Case of Dermassist

In 2020, scientists at Google published results from a skin disease algorithm, which could diagnose skin conditions, including melanoma, to a standard that was noninferior to dermatologists and that was superior to that of a GP or nurse.^24^ In 2021, Google’s algorithm was used to power the DermAssist smartphone app, which was released to patients of all skin tones in Europe and granted a CE mark as a Class I medical device in the European Union.^25^ It is unclear if the DermAssist app will remain available in the European Union after 2025, when it would need to comply with more rigorous regulatory standards that do not rely on self-certification.^26^ The DermAssist app has also not received regulatory approval in the United States.^27^

The DermAssist app has come under criticism for its portrayal by Google as a search tool, rather than a medical diagnostic device, which patients might reasonably rely on to make life and death health decisions.^28^ There have also been concerns that in an attempt to provide safeguards against false-negative results—such as incorrectly indicating that a lesion is not melanoma—the app developers may have been willing to accept much higher rates of false-positive results.^29^ More false positives, in turn, have the potential to needlessly worry patients, as well as to flood already-stretched doctors’ offices with a ‘tsunami of overdiagnosis’.^30^ Most pertinently, the model underlying DermAssist was also criticised for being trained and validated using data heavily skewed towards lighter-skinned patients. For Type VI skin on the Fitzpatrick scale (which is the darkest), only 46 samples (out of 16,530) were used for algorithmic training. Similarly, only 1 Type VI sample (out of 4,146) was used for algorithmic validation.^31^ These sparse dark-skinned samples were used to support diagnoses across 26 different skin conditions, which means that for many skin conditions there were no Type VI samples used for either training or validation purposes.^32^ Given the sparsity of training data, there are justifiable concerns that this could lead to increased algorithmic errors for dark-skinned patients, as well as to disproportionally assigning false-positive results in an effort to limit life-threatening false-negative diagnoses.

There is not enough publicly available information to assess the extent to which the DermAssist app has been beneficial or harmful for dark-skinned melanoma patients. While Google indicated that additional data were gathered before DermAssist was launched,^33^ the company also acknowledged that a lack of data from dark-skinned patients is a problem that the whole field of dermatology continues to suffer from.^34^ There is also no public information regarding the real-world performance of the DermAssist app, nor has the app itself been subject to a prospective clinical trial. Within this information vacuum and within the limited scope of this article, it is not possible to assess whether it was appropriate for Google to have launched the DermAssist app in 2021, particularly with respect to dark-skinned melanoma patients. We will, however, focus on a more narrow, hypothetical question: if the DermAssist app was in fact beneficial to light-skinned melanoma patients, but not beneficial, and even harmful (e.g., if it produced false negatives), for dark-skinned melanoma patients, should the app have been selectively offered to only light-skinned users, or should the app’s release have been delayed for everyone until all patient groups would benefit?

Even before we factor in the implications of historic injustice, there is a general presumption that health services should be developed for (and available to) all groups within society—rich and poor, young and old, men and women, light-skinned and dark-skinned.^35^ This presumption is behind the universal healthcare systems that are a feature of most wealthy countries, as well as in the concepts of beneficence and justice, which represent foundational elements within the field of bioethics.^36^

Despite there being a presumption that health services should be generally available, we argued in the male breast cancer case study that it is ethically acceptable to override this presumption if there are countervailing ethical considerations that are sufficiently compelling. These compelling considerations, which related to utility, included the large number of women that could be helped, as well as the relatively small number of men involved—who would not be made worse off if they were denied algorithmic access, but who might suffer harm if they were exposed to inadequately trained algorithmic approaches. These countervailing ethical considerations also apply in the DermAssist case study, which brings with it a further set of countervailing issues relating to historic injustice.

Before addressing historic injustice, it is worth noting one important difference between the Google and Cambridge examples. There is a basic ethical principle of ‘ought implies can’. Google is a $1.5 trillion company. One might reasonably assume that they have the financial means to get representative samples. Cambridge researchers were operating on a relatively miniscule budget and questions of distributive justice and the best use of their limited resources arise. But let us assume, for argument’s sake, that there are practical obstacles to quickly obtaining representative data that cannot be overcome by simply spending more money.

Should historic injustice override all other ethical considerations, including the lives of people who could be helped, as outlined above? Or should historic injustice be weighed against the other ethical considerations that have thus far been presented?

For example, is DermAssist analogous to selectively denying other forms of health care, such as emergency room care? Denying emergency room care to certain groups is of course a totally unacceptable and blatant form of racism. Would a light-skinned-only melanoma algorithm be any different? There is a difference. Emergency room care is an issue related to access rights. By simply making a policy decision, one can grant (or deny) access to emergency room care to dark-skinned people. One cannot, however, simply mandate that dark-skinned patients have access to a well-performing melanoma diagnostic algorithm—because the data (and indeed the knowledge) to create such an algorithm for dark-skinned patients may not actually exist and may take years to develop, even if sufficient resources are deployed. We call this a scientific reason for the difference. However, we note that there is not a simple distinction between the two. For example, the data may not exist because insufficient effort has been put into reaching those populations, in the same way that emergency room access may be officially available but in fact be limited by the chosen locations of the facilities, for example.

Indeed, contributing factors to this knowledge deficit may relate to issues of historic injustice. Historic injustice may mean that there is even less dark-skinned melanoma data than would be expected, purely based on the comparative rarity of melanoma in dark-skinned patients.^37^ Dark-skinned patients generally have less access to health care, lower levels of trust in the healthcare system, and lower levels of participation rates in medical research, as compared to light-skinned patients.^38^ These factors would all be expected to increase the size of the epistemic deficit in relation to dark-skinned patients. This epistemic deficit may have also been exacerbated by wrongful actions, including outright racism, structural racism and general research hesitancy brought about by research abuses, such as the Tuskegee syphilis experiments.^39^ It may also be the case that current healthcare disparities—perhaps, once again, brought about by historic injustice—mean that dark-skinned melanoma sufferers are particularly in need of algorithmic services.

None of these factors change the fact that an epistemic gap may nevertheless exist, which prevents the deployment of an accurate algorithmic diagnostic for dark-skinned melanoma patients. This means that the Expedited Deployment option is unethical. To launch an algorithm for all patient groups in this context, simply because it works well for light-skinned patients, would effectively sacrifice dark-skinned patient welfare by making these patients relatively and absolutely worse off through inaccurate diagnoses. Accepting harm to dark-skinned patients as a form of ‘collateral damage’ in the pursuit of light-skinned benefits would run counter to deontological traditions within ethics and to widely accepted norms within bioethics, while also further eroding trust within dark-skinned communities.^40^

There are therefore two options remaining: Delayed Deployment and Selective Deployment. Delayed Deployment requires us to disregard the interests of light-skinned melanoma patients, who researchers do have the knowledge to help, even though this asymmetric epistemic position may have been brought about (and exacerbated by) the historic injustice against dark-skinned patients.

We believe that the ethical tensions arising from the delayed and expedited deployment options are sometimes best resolved through a selective deployment approach; this approach is not ideal, but instead—with appropriate regulation—represents the best way to balance harm prevention, utility and fairness considerations.

Selective deployment, we believe, is justified in the breast cancer case for two reasons. First, men have not been victims of historical injustice. Second, expedited deployment would harm men by recommending inappropriate treatment options.

What of the melanoma case? The answer will depend on the level of harm which would result from deploying a less reliable algorithm on the unrepresented population. In the case of DermAssist, it appears that the sensitivity and specificity thresholds have been set to create high false positives amongst dark skinned patients. If this does lead to greater specialist attendance, and higher rates of effective treatment, the algorithm may still be beneficial overall to dark-skinned people (despite creating some unnecessary expenditure and anxiety). In this case, it should be generally deployed but with accurate group-specific information about sensitivity and specificity.

However, if the deployment of the DermAssist app would cause more overall harm than benefit to dark-skinned groups, there is a strong argument for selective deployment to light- skinned groups while more data is urgently gathered.

Selective algorithmic deployment should not, however, be done through stealth. Instead, system developers should be transparent with stakeholders regarding the reasons behind selective deployment, as well as the path towards greater inclusivity in the future. Being clear about the rationale behind selective deployment also plays a critical role in protecting the dignity of excluded populations, who deserve to know the reasons why they have not been included in algorithmic approaches and when they can expect this situation to be rectified. Without this sort of transparency, system developers cannot be held to account for either their short-term implementation approach or their medium-term commitments to increase inclusivity.

Indeed, selective algorithmic deployment should be a short-term solution. Rectifying epistemic gaps for disadvantaged patient groups should be a priority within the field of health care, as this will help to move towards universally deployed algorithmic approaches, which work well for all patient groups and can be leveraged to improve health equity. This means that further data gathering for disadvantaged groups should be expedited—including in clinical research settings, which can be an important route for new data generation. As algorithmically excluded groups will need to have greater reliance on human interventions, greater efforts should also be made to improve the training provided to healthcare professionals, so that they are better placed to assess health conditions within diverse populations.

In the meantime, efforts should be made to lower the barriers for nonalgorithmic interventions, such as diagnoses made by healthcare professionals, which are currently seen as the gold standard for patient care in many domains. Access to nonalgorithmic diagnostics should be streamlined, simplified and subsidised—linking seamlessly into down-stream treatment options. Careful consideration should also be given to the merits of increasing the sensitivity of diagnostic assessments for understudied groups, although this would require increased resource utilisation, it would help to reduce dangerous false-negative tests results, though at the cost of increased resource utilisation, needless worry on the part of patients, and unnecessary medical procedures, which may carry some degree of risk.

## 6 Potential Objections

A potential objection to our argument is that it is simply not appropriate for light-skinned patients to receive health benefits before dark-skinned patients, who are already worse off. If health equity is a key component of justice, and if ‘justice delayed is justice denied’, then our proposals could be viewed as unjust, and therefore ethically unacceptable.

If the weight of this critique rests on the unacceptability of delayed healthcare benefits for some groups, then a supporter of this critique may also need to ‘bite the bullet’ and accept that tens of thousands of women should be denied the benefits of the Cambridge breast cancer algorithm, as a small number of men are currently unable to benefit from similar algorithmic approaches. We do not believe that most people would be willing to accept this type of ‘levelling down’ conclusion.

To avoid biting this bullet, the critique could be modified to say that delaying men’s access to the algorithm is ethically acceptable, but that delaying benefits to a group that has been subjected to historic injustice is unacceptable, primarily because this delay would compound disadvantage. Not all men are, however, advantaged. Indeed, while all men are susceptible to breast cancer, men of African descent—who have been subjected to historic injustice—are proportionally more likely to suffer from the disease than white men.^41^ Along this line of reasoning, it could also be argued that more advantaged women should be denied beneficial algorithms, as long as similar algorithmic tools are not available to male breast cancer sufferers, particularly those of African descent. We believe this would also be an ethically untenable conclusion.

To avoid biting this bullet, the critique could be modified a third time—by ignoring the inter-sectional tensions outlined above. As women (as a group) are generally more disadvantaged than men (as a group), it could be argued that it is right to give women priority over men, thereby making it acceptable to release the Cambridge algorithm for women before men. However, as dark-skinned patients (as a group) are generally more disadvantaged them light skinned patients (as a group), it could be argued that it would be wrong to give light-skinned patients priority over dark-skinned patients, making it ethically unacceptable to release DermAssist for light-skinned patients before dark-skinned patients.

Faced with these objections, in the examples above, we would agree that women should be given priority over men, and that dark-skinned patients should be given priority over light-skinned patients. The basis of this priority is that women and dark-skinned patients are (generally) more disadvantaged than men and light-skinned patients. But how *much* priority should be given?

In his paper ‘Equality and Priority’, Derek Parfit puts forward the Priority View—which stipulates that benefits to the worse off should be given greater priority, but that this priority should not be absolute, particularly if it would undermine sufficiently large benefits for better off groups.^42^ Parfit’s Priority View aims to strike a balance between more extreme egalitarian and utilitarian positions—by providing a coherent ethical framework for rejecting unpalatable levelling-down scenarios advocated by scholars such as Temkin (who can give too much priority to those that are worse off), while also injecting greater equity into purely utilitarian frameworks advocated by scholars such as Harsanyi (who can give too little priority to those that are worse off).^43^

In the Cambridge example, we believe that an appropriate amount of priority is extended to women, who were able to benefit from the algorithm, which for legitimate reasons was not available to men, who were nevertheless protected from algorithmic harm. We would also accept that the circumstances involved in the dark-skinned melanoma example are more challenging, with potentially fewer legitimate reasons why the relevant darker-skinned data were not available and why dark-skinned patients are generally worse off than light-skinned patients. In our view, however, these circumstances need to be balanced against what Parfit might describe as the ‘sufficiently large benefits’ that would need to be denied to light-skinned patients—who suffer from melanoma at a much higher rate of incidence than patients with dark skin. Instead, we have argued that priority for patients with dark skin may be best achieved through a basket of other nonalgorithmic interventions, which we have discussed above and which we will shortly summarise in the conclusion below. (Of course, this argument hinges on DermAssist actually providing sufficiently large benefits to light skinned patients that could not otherwise be reasonably achieved. That is at present postulated, but not proven).

While this article has only addressed two case studies, we can give some indication as to where the boundary conditions might lie for our approach. We believe that the bar for selective deployment should be lower when exclusionary algorithms will not lead to incremental harms for excluded populations, when it is possible to deliver appropriate nonalgorithmic services to excluded populations and when it is not possible to collect incremental data for excluded population within the short/medium term—as would be the case for rare conditions, when historic data has been collected in clinical trials or when longitudinal datasets are involved. To the extent that these conditions do not hold, the ethical case for moving forward with exclusionary algorithms is made much more challenging, particularly in light of aggravating circumstances, such as historic discrimination.

**Figure 1:**
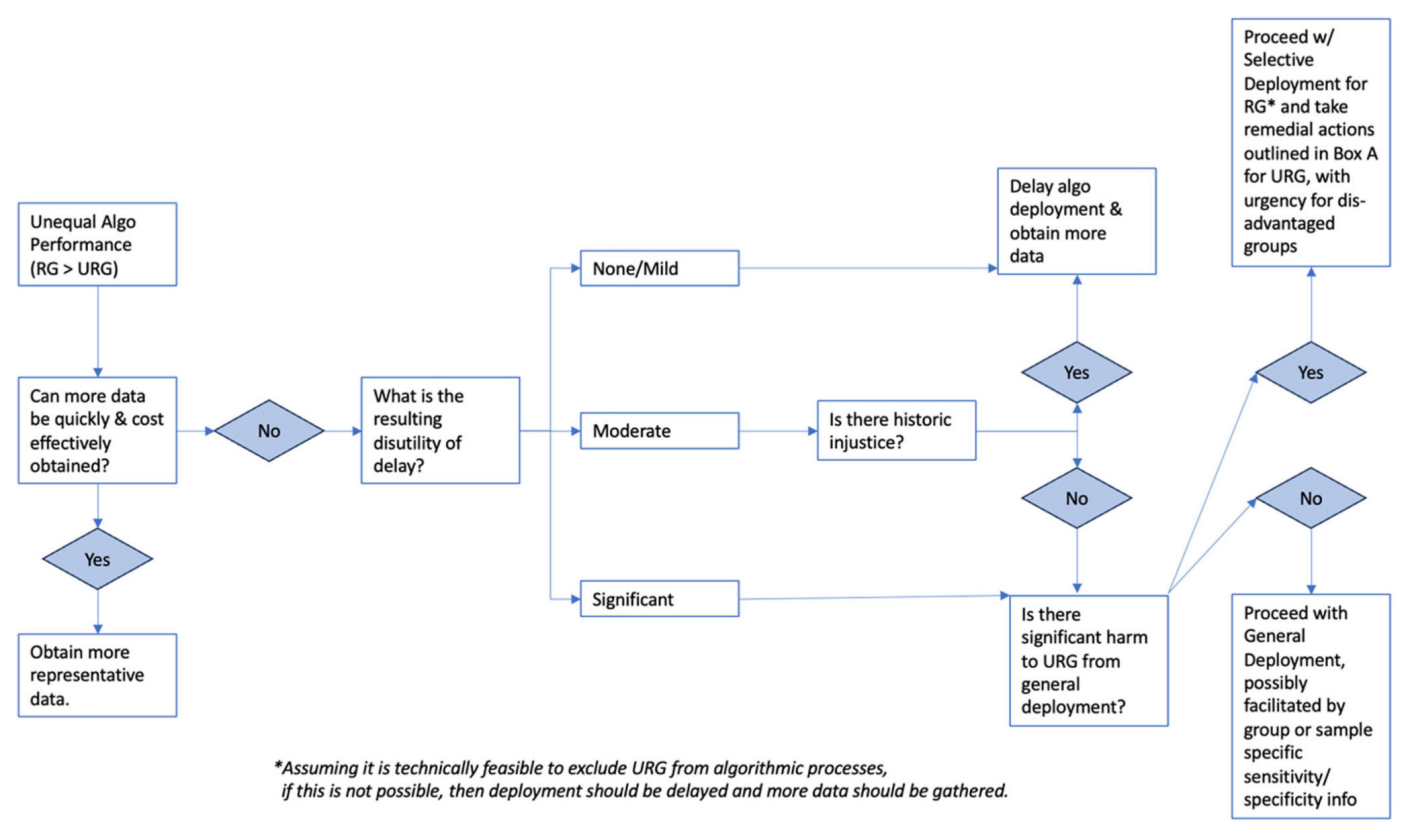
An ethical algorithm for the general and selective deployment of unrepresentative AI in health care. RG, represented group; URG, unrepresented group.

That being said, the main purpose of this article is to make the conceptual point, which is somewhat novel in the field of AI ethics, that there are some circumstances where it can be ethically acceptable to exclude some populations (and even historically disadvantaged populations) from algorithmic approaches. Further research is urgently needed to explore the limits of this conceptual approach in a broader variety of case studies. It is, however, beyond the scope of this article to fully resolve where all of these lines should be drawn—in the same way that questions of proportionality, ‘small sacrifice’ and ‘easy rescue’ are all open questions within the field of practical ethics.^44^ We have summarised the relevant factors which need to be more deeply interrogated in Figure 1.

Another potential objection to our arguments is that in some cases the selective deployment of algorithmic approaches could mean that deselected groups may never be included—if their conditions are sufficiently rare or if it is not sufficiently beneficial (in cost/benefit terms) to bring these populations into algorithmic processes. How could such a result be justified, particularly for disadvantaged groups, which we have argued should be given priority?

With respect to small populations, it may very well be the case that these groups are never served by algorithmic systems. At the extreme end of the spectrum, we can think about *n* = 1 populations or deep precision medicine. For these individuals, their main risk is that they are *inadvertently* served by algorithmic processes that cannot cope with their unique attributes, leading to potentially unsafe algorithmic outputs. In these cases, as safe inclusion may not be possible, the main ethical imperative is to prevent unsafe inclusion—with priority viewed in terms of making incremental efforts to avoid this type of harm, rather than accepting it as a form of ‘collateral damage’ in the pursuit of incremental benefits for better represented populations. How AI can best serve individuals in deep precision medicine is beyond the scope of this article.

With respect to the question of cost/benefit analyses, it would not be surprising if incorporating excluded groups into algorithmic processes is more expensive (per person) than including groups that are already well-represented in existing data. A key aspect of showing priority to excluded populations is to nevertheless move forward with inclusive measures (such as undertaking additional data collection efforts) even when there are diminishing marginal returns for doing so. There may be a role for government support where there are genuine barriers to private enterprises in achieving representation in excluded populations (though this does not appear to be the case with DermAssist app).

The greater the level of disadvantage that has been suffered by a particular group which has been temporarily excluded from an algorithmic process, the greater the level of priority that group should be afforded in terms of pressing forward with its eventual inclusion and the lower the level of marginal benefit that should be required to justify seeking a more inclusive outcome. As discussed above, however, there will also come a point for very small populations where there is not a sufficient critical mass of data generation opportunities to support robust algorithmic implementation—which may result in the need to offer nonalgorithmic services to these groups, even in the long run.

## 7 A Roadmap for Better Research

One major practical problem with this approach is that it may reduce the incentive for companies to build algorithms ethically in the first place. If there is an option to produce a ‘majority’ version of a product with less investment of resources than would be required to create the product for all populations, and only the need for a vague promise of future versions, it may encourage even less attention to minority groups in the development phase. It is important then that there are regulatory measures to ensure that (1) there is a good scientific reason behind the layered roll out; (2) the plan to develop the algorithm for minority populations is appropriate and carried out in a timely manner. Moreover, it may be helpful to provide government support to promote the development of services for minority groups where it is genuinely not feasible in a private setting.

It is important to note that the status quo is not a neutral option. The DermAssist case study shows the potential for current products to nontransparently underperform for minority groups, with attendant risks of poorer quality care to groups that already face historic injustice.

## 8 Limitations

The examples used in this article involve rare conditions for those patients that might be algorithmically excluded—such as men with breast cancer and dark-skinned melanoma sufferers. Further research would need to be undertaken with respect to the ethical calculus behind selective algorithmic deployments associated with more common conditions, such as diabetes or heart disease. Indeed, in this article, we have only explored two case studies, both of which had somewhat unusual fact patterns with respect to those patients that were underrepresented in algorithmic training data. Caution, therefore, should be applied when attempting to extrapolate our conclusions to other case studies—and further research should be undertaken to test the limits of the theoretical approach that we develop in this article, with the view to better understanding the conditions under which considerations of equity would override considerations of utility.

The arguments in this article are also premised on the ability to meaningfully differentiate between subgroups, which enables exclusion (and thereby protection) of underrepresented subgroups from algorithmic approaches. Underrepresented subgroups can, however, be subtle, complex, multidimensional, or intersectional—and most importantly not falling along ‘traditional’ group boundaries such as race, sex, and so on—making it difficult to accurately differentiate between various subgroups in practice. To the extent that meaningful subgroups are nonexcludable from algorithmic approaches through human processes, similar results could in theory be achieved using algorithmic tools—such as out of distribution (OOD) detectors (which are used to identity samples that are outside of the data distribution that was used in algorithmic training data) and uncertainty estimations (which aim to place sample-specific confidence intervals around algorithmic predictions).^45^ In practice, however, the cited technical literature has questioned the reliability of these approaches for machine-learning models in many domains, highlighting the potential difficulties of relying on these methods in high-stakes applications.^46^ If workable approaches for algorithmic exclusion are not possible in a particular therapeutic area, our view is that this would point towards delaying algorithmic deployment until the technology worked reasonably well for underrepresented groups—who could otherwise be made even worse off at the expense of making privileged groups even better off.

Alternativity, rather than excluding underrepresented groups, these populations could instead be given access to tailored sensitivity/specificity information to help them make informed decision on the algorithmic predictions that they receive—even in circumstances where better represented groups have access to more accurate predictions. This approach would require that clinically useful levels of accuracy can be achieved for under-represented groups and that algorithmic systems are capable of reliably producing robust sensitivity/specificity information within sparse data environments. Understanding the ethical and practical implications of taking a group-specific (or sample-specific) sensitivity/specificity approach to algorithmic access is an area that we are actively researching.

Even if there is a workable technical solution for enabling algorithmic exclusion in some therapeutic areas, further research is required to better understand the practical and ethical implications of potentially having a large patchwork of intersectional groups that could be excluded from algorithmic services. Systematic reviews of intersectionality in health care highlight the limited progress that has been made in implementing workable solutions to intersectional issues, either in healthcare research or in clinical applications.^47^ In the case of breast cancer prognostics, it may not only be men who could be excluded from algorithmic approaches—but also trans women, very young women and other relatively rare breast cancer patient groups, all of whom might suffer from various data scarcity issues. Determining where to draw the line on how much inclusion should be required to justify an algorithmic implementation (as well as what level of algorithmic performance should be required for each group to be included in an algorithmic process) are open research questions—though lessons could no doubt be learned from other areas where these issues have already been faced, such as in health technology assessment exercises or in national health screening programmes.

As noted in Section 1, at the beginning of this article, there is also a considerable gap between the future promise of algorithmic approaches and their ability to deliver safe and effective results in the clinic today. Bridging this gap will not only require dealing with the algorithmic exclusion issues raised in this article but also a host of other issues that have already been raised within the algorithmic literature, including concerns over system validation, robustness, generalisability, scalability, transparency, safety, regulation and integration into clinical workflows—at system launch, as well as over time, when real-world conditions may drift from the original algorithmic training data.^48^ Indeed, given the vast array of issues that need to be resolved before algorithmic systems can be ethically deployed in healthcare settings, temporarily excluded populations can benefit from being ‘fast followers’ in future releases of algorithmic systems that are initially put in place for better represented populations. In being shielded from the first wave of algorithmic deployments, taking a ‘fast follower’ approach could also help to allay fears in some marginalised communities, which can suffer from research hesitancy, distrust in medical professionals and a reluctance to take advantage of healthcare innovations.^49^ Moreover, proving that algorithmic approaches can be beneficial for participants in the first wave of algorithmic deployments will increase the urgency with which data is collected to help increase the inclusiveness of subsequent deployments.

Though there is limited information available on the inner workings of the DermAssist app, one step that Google appears to have taken to address potential system shortcomings is to refer questionable images to a human pathologist for review before readouts are given to app users.^50^ While it is not clear whether these internal referrals are based on OOD detectors, uncertainty estimations, or patient profiling, such an approach—if robust—could be an elegant and seamless way of delivering more equitable services to underrepresented groups, as well as creating a new avenue for diverse data collection. The extensive use of human referrals for underrepresented groups in the DermAssist app should not, however, be confused with the universal provision of *algorithmic* services. Indeed, such an approach is more reminiscent of the Mechanical Turk, which was a ‘machine’ constructed in 1770 that appeared to beat human opponents at chess, when it was in fact simply a human hiding in a box pretending to be a chess-playing algorithm^51^—raising questions about both transparency and scalability.

Finally, this article focuses on the ethical issues associated with the selective deployment of algorithms. As algorithmic exclusion criteria might rely on (or indirectly impact) protected characteristics, the current legal and regulatory environment in many jurisdictions may favour universal algorithmic deployments—even if this results in delayed deployments for everyone or in harmful deployments for those who could have been better protected through more exclusionary approaches. Further research linking the ethical issues explored in this article to the broader legal and regulatory environment should be pursued on an urgent basis, to ensure that the best outcomes can be achieved for patients in the rapidly evolving field of AI-enabled diagnostic and prognostic tools.

## 9 Conclusion

We have argued against deploying diagnostic and prognostic algorithms for groups that are underrepresented in algorithmic training data and who could therefore be subjected to algorithmic harm. Instead, we have argued in favour of selectively deploying algorithmic approaches for female breast cancer patients and, in some circumstances, for light-skinned melanoma patients, even though this meant that underrepresented groups would not immediately benefit from algorithmic advances.

While dark-skinned melanoma patients suffer from historic injustice, we argued that, so long as care for those patients remains unchanged, and in so far as they would be harmed by general deployment, historic injustice does not justify levelling down potential benefits for light-skinned melanoma patients, who would needlessly suffer if algorithmic deployment was delayed for all patients until more equi results could be achieved. While the context of historic injustice can pose considerable challenges for the ethical acceptability of selective algorithmic deployment strategies, for the case studies addressed in this article, we have argued that the issue of historic injustice is better addressed by implementing a broad range of nonalgorithmic interventions, which are summarised below in Box 1. We have identified the relevant factors which need to be considered in deciding whether to generally deploy, delay or selective deploy algorithms in health care. We have created an ethical algorithm or decision procedure for making these decisions, which is outlined in Figure 1.

In exploring the case studies, we also noted some of the ways in which selective algorithmic deployment could be achieved in practice—including through the use of manual patient segmentation, OOD detectors and uncertainty estimations—noting the potential robustness issues with each of these approaches. Finally, we also discussed the potential benefits of seamlessly embedding nonalgorithmic services for excluded groups into algorithmic and clinical workflows, while noting transparency and scalability concerns.

## Box 1 Interventions for algorithmically excluded communities

- Greater transparency for impacted communities.
- Increased data collection efforts.
- Increased inclusion in clinical research.
- Increased training for healthcare professionals.
- Increased provision of nonalgorithmic services.
- Reduced access barriers for human interventions.
- Reduced access barriers for downstream therapeutic interventions.
- Seamless integration with algorithmic services.
- Identified pathway for universal algorithmic deployment.

## Footnotes

1 Goldenberg, S. L., Nir, G., & Salcudean, S. E. (2019). A new era: Artificial intelligence and machine learning in prostate cancer. *Nature Reviews Urology, 16*(7), 391–403. https://doi.org/10.1038/s41585-019-0193-3

2 Andaur Navarro, C. L., Damen, J. A. A., Takada, T., Nijman, S. W. J., Dhiman, P., Ma, J., Collins, G. S., Bajpai, R., Riley, R. D., Moons, K. G. M., & Hooft, L. (2023). Systematic review finds “spin” practices and poor reporting standards in studies on machine learning-based prediction models. *Journal of Clinical Epidemiology, 158*, 99–110. https://doi.org/10.1016/j.jclinepi.2023.03.024

3 Liu, X., Faes, L., Kale, A. U., Wagner, S. K., Fu, D. J., Bruynseels, A., Mahendiran, T., Moraes, G., Shamdas, M., Kern, C., Ledsam, J. R., Schmid, M. K., Balaskas, K., Topol, E. J., Bachmann, L. M., Keane, P. A., & Denniston, A. K. (2019). A comparison of deep learning performance against health-care professionals in detecting diseases from medical imaging: A systematic review and meta-analysis. *Lancet Digital Health, 1*(6), e271–e297. https://doi.org/10.1016/S2589-7500(19)30123-2

4 D’Amour, A., Heller, K., Moldovan, D., Adlam, B., Alipanahi, B., Beutel, A., Chen, C., Deaton, J., Eisenstein, J., Hoffman, M. D., Hormozdiari, F., Houlsby, N., Hou, S., Jerfel, G., Karthikesalingam, A., Lucic, M., Ma, Y., McLean, C., Mincu, D., … Sculley, D. (2022). Underspecification presents challenges for credibility in modern machine learning. *Journal of Machine Learning Research, 23*(1), 10237–10297.

5 Steiner, D. F., Chen, P. C., & Mermel, C. H. (2021). Closing the translation gap: AI applications in digital pathology. *Biochimica et Biophysica Acta. Reviews on Cancer, 1875*(1), 188452. https://doi.org/10.1016/j.bbcan.2020.188452; Roberts, M., Driggs, D., Thorpe, M., Gilbey, J., Yeung, M., Ursprung, S., Aviles-Rivero, A. I. Etmann, C., McCague, C., Beer, L., Weir-McCall, J. R., Teng, Z., Gkrania-Klotsas, E., AIX-COVNET, Rudd, J. H. F., Sala, E., & Schönlieb, C. (2021). Common pitfalls and recommendations for using machine learning to detect and prognosticate for COVID-19 using chest radiographs and CT scans. *Nature Machine Intelligence, 3*(3), 199–217. https://doi.org/10.1038/s42256-021-00307-0

6 Harwell, D. (2020, December). Algorithms are deciding who gets the first vaccines. Should we trust them? *The Washington Post*; Ledford, H. (2019). Millions of black people affected by racial bias in health-care algorithms. *Nature, 574*(7780), 608–609. https://doi.org/10.1038/d41586-019-03228-6

7 Kleinberg, J. (2018). *Inherent trade*-*offs in algorithmic fairness*. https://doi.org/10.1145/3219617.3219634; Barocas, S. (2023). *Fairness and machine learning*. MIT Press.

8 Giovanola, B., & Tiribelli, S. (2023). Beyond bias and discrimination: Redefining the AI ethics principle of fairness in healthcare machine-learning algorithms. *AI and Society, 38*(2), 549–563. https://doi.org/10.1007/s00146-022-01455-6; Grote, T., & Keeling, G. (2022). On algorithmic fairness in medical practice. *Cambridge Quarterly of Healthcare Ethics, 31*(1), 83–94. https://doi.org/10.1017/S0963180121000839; Fazelpour, S., & Danks, D. (2021). Algorithmic bias: Senses, sources, solutions. *Philosophy Compass, 16*(8), e12760. https://doi.org/10.1111/phc3.12760

9 American Cancer Society. (2023). *Cancer facts and figures 2023*. American Cancer Society.

10 Ferzoco, R. M., & Ruddy, K. J. (2016). The epidemiology of male breast cancer. *Current Oncology Reports, 18*(1), 1. https://doi.org/10.1007/s11912-015-0487-4

11 Yalaza, M., İnan, A., & Bozer, M. (2016). Male breast cancer. *Journal of Breast Health, 12*(1), 1–8.

12 American Cancer Society. (n.d.). *Melanoma skin cancer statistics*. https://www.cancer.org/cancer/melanoma-skin-cancer/about/key-statistics.html

13 Ferzoco & Ruddy, op. cit. note 10.

14 Hassett, M. J., Somerfield, M. R., & Giordano, S. H. (2020). Management of male breast cancer: ASCO guideline summary. *JCO Oncology Practice, 16*(8), e839–e843. https://doi.org/10.1200/JOP.19.00792

15 Gucalp, A., Traina, T. A., Eisner, J. R., Parker, J. S., Selitsky, S. R., Park, B. H., Elias, A. D., Baskin-Bey, E. S., & Cardoso, F. (2019). Male breast cancer: A disease distinct from female breast cancer. *Breast Cancer Research and Treatment, 173*(1), 37–48. https://doi.org/10.1007/s10549-018-4921-9

16 Alaa, A. M., Gurdasani, D., Harris, A. L., Rashbass, J., & van der Schaar, M. (2021). Machine learning to guide the use of adjuvant therapies for breast cancer. *Nature Machine Intelligence, 3*(8), 716–726. https://doi.org/10.1038/s42256-021-00353-8; Despite the large volume electronic health record data that can support prognostic algorithms of this type, there is limited supporting evidence for claims that these algorithms outperform clinicians—who do not routinely predict risk in this way, or track the predictions that they do make in a way that can be robustly compared with algorithmic outputs.

17 Ibid.

18 The Fitzpatrick Scale itself has been criticised for its disproportionate focus on white skin tones. See, for example, Okoji, U. K., Taylor, S. C., & Lipoff, J. B. (2021). Equity in skin typing: Why it is time to replace the Fitzpatrick scale. *British Journal of Dermatology, 185*(1),198–199. https://doi.org/10.1111/bjd.19932.

19 Mahendraraj, K., Sidhu, K., Lau, C. S. M., McRoy, G. J., Chamberlain, R. S., & Smith, F. O. (2017). Malignant melanoma in African–Americans: A population-based clinical outcomes study involving 1106 African-American patients from the surveillance, epidemiology, and end result (SEER) database (1988–2011). *Medicine, 96*(15), e6258. https://doi.org/10.1097/MD.0000000000006258

20 Hu, S., Soza-Vento, R. M., Parker, D. F., & Kirsner, R. S. (2006). Comparison of stage at diagnosis of melanoma among Hispanic, black, and white patients in Miami-dade county, Florida. *Archives of Dermatology, 142*(6), 704–708. https://doi.org/10.1001/archderm.142.6.704

21 Adamson, A. S., & Smith, A. (2018). Machine learning and health care disparities in dermatology. *JAMA Dermatology, 154*(11), 1247–1248. https://doi.org/10.1001/jamadermatol.2018.2348

22 Bradford, P. T. (2009). Skin cancer in skin of color. *Dermatology Nursing, 21*(4), 170–178.

23 Gloster, H. M., & Neal, K. (2006). Skin cancer in skin of color. *Journal of the American Academy of Dermatology, 55*(5), 741–60; quiz 761. https://doi.org/10.1016/j.jaad.2005.08.063

24 Liu 2020.

25 Murgia, M. (2021). Google launches AI health tool for skin conditions. *Financial Times*.

26 Simonite, T. (2021, June 23). Google launches a new medical app—Outside the US. *Wired*.

27 Ibid.

28 Ibid.

29 Davey, M. (2021). Doctors Fear google skin check app will lead to “tsunami of overdiagnosis.” *The Guardian*.

30 Ibid.

31 Liu, op. cit. note 22.

32 Ibid.

33 Murgia, op. cit. note 25.

34 Feathers, T. (2021). Google’s new dermatology app wasn’t designed for people with darker skin *Motherboard*.

35 Delamothe, T. (2008). Founding principles. *BMJ, 336*(7655), 1216–1218. https://doi.org/10.1136/bmj.39582.501192.94

36 Beauchamp, T., & Childress, J. (2009). *Principles of biomedical ethics*. Oxford University Press.

37 Adamson & Smith, op. cit. note 21.

38 Sirugo, G., Williams, S. M., & Tishkoff, S. A. (2019). The missing diversity in human genetic studies. *Cell, 177*(4), 1080. https://doi.org/10.1016/j.cell.2019.04.032

39 Freimuth, V. S., Quinn, S. C., Thomas, S. B., Cole, G., Zook, E., & Duncan, T. (2001). African Americans’ views on research and the tuskegee syphilis study. *Social Science and Medicine, 52*(5), 797–808. https://doi.org/10.1016/s0277-9536(00)00178-7

40 Beauchamp & Childress, op. cit. note 36.

41 Ferzoco & Ruddy, op. cit. note 9.

42 Parfit, D. (1997). Equality and priority. *Ratio, 10*(3), 202–221.

43 Temkin, L. S. (2003). Equality, priority or what? *Economics and Philosophy, 19*(1), 61–87. https://doi.org/10.1017/S0266267103001020; Harsanyi, J. C. (1975). Can the maximin principle serve as a basis for morality? A critique of John Rawls’s theory. *American Political Science Review, 69*(2), 594–606. https://doi.org/10.2307/1959090

44 Cameron, J., Stewart, C., & Savulescu, J. (2021). Assessing rationing decisions through the principle of proportionality. *Journal of Law Medicine, 28*(4), 955–964.

45 Nado, Z. (2021). Uncertainty baselines: Benchmarks for uncertainty and robustness in deep learning *Arxiv;* Yang, J. (2022). OpenOOD: Benchmarking generalised out-of-distribution detection *Arxiv*; Salehi, M. (2021). A unified survey on anomaly, novelty, open-set, and out-of-distribution detection: Solutions and future challenges *Arxiv*; Gawlikowski, J., Tassi, C. R. N., Ali, M., Lee, J., Humt, M., Feng, J., Kruspe, A., Triebel, R., Jung, P., Roscher, R., Shahzad, M., Yang, W., Bamler, R., & Zhu, X. X. (2021). A survey of uncertainty in deep neural networks. *Artificial Intelligence Review*. https://doi.org/10.1007/s10462-023-10562-9

46 Goetz L, Seedat N, Vandersluis R, van der Schaar M (2024). Generalisation—A key challenge for responsible AI in patient-facing clinical applications. *Nature Machine Intelligence*. 2024

47 Tinner, L., Holman, D., Ejegi-Memeh, S., & Laverty, A. A. (2023). Use of intersectionality theory in interventional health research in high-income countries: A scoping review. *International Journal of Environmental Research Public Health, 15*, 20(14):6370. https://doi.org/10.3390/ijerph20146370

48 Steiner, D. F., Chen, P-H. C., Mermel, & C. H. (2021). Closing the translation gap: AI applications in digital pathology. *BBA*—*Reviews on Cancer, 1875*(2021), 188452.

49 Martin, K. J., Stanton, A. L., & Johnson, K. L. (2023). Current health care experiences, medical trust, and COVID-19 vaccination intention and uptake in Black and White Americans. *Health Psychology, 42*(8), 541–550. https://doi.org/10.1037/hea0001240

50 Murgia, op. cit. note 24.

51 Schaffer, S. (1999). Enlightened automata. In W. Clark, J. Golinski, & S. Schaffer (eds.), *The sciences in enlightened Europe* (pp. 126–165). The University of Chicago Press.

